# Asprosin in the Paraventricular Nucleus Induces Sympathetic Activation and Pressor Responses via cAMP-Dependent ROS Production

**DOI:** 10.3390/ijms232012595

**Published:** 2022-10-20

**Authors:** Xiao-Li Wang, Jing-Xiao Wang, Jun-Liu Chen, Wen-Yuan Hao, Wen-Zhou Xu, Zhi-Qin Xu, Yu-Tong Jiang, Pei-Qi Luo, Qi Chen, Yue-Hua Li, Guo-Qing Zhu, Xiu-Zhen Li

**Affiliations:** 1 Key Laboratory of Targeted Intervention of Cardiovascular Disease, Collaborative Innovation Center of Translational Medicine for Cardiovascular Disease, Department of Physiology, Nanjing Medical University, Nanjing 211166, China; 2 Department of Cardiology and Emergency Department, The Second Affiliated Hospital of Nanjing Medical University, Nanjing 210011, China; 3 Department of Pathophysiology, Nanjing Medical University, Nanjing 211166, China

**Keywords:** asprosin, paraventricular nucleus, reactive oxygen species, sympathetic activity, blood pressure

## Abstract

Asprosin is a newly discovered adipokine that is involved in regulating metabolism. Sympathetic overactivity contributes to the pathogenesis of several cardiovascular diseases. The paraventricular nucleus (PVN) of the hypothalamus plays a crucial role in the regulation of sympathetic outflow and blood pressure. This study was designed to determine the roles and underlying mechanisms of asprosin in the PVN in regulating sympathetic outflow and blood pressure. Experiments were carried out in male adult SD rats under anesthesia. Renal sympathetic nerve activity (RSNA), mean arterial pressure (MAP), and heart rate (HR) were recorded, and PVN microinjections were performed bilaterally. Asprosin mRNA and protein expressions were high in the PVN. The high asprosin expression in the PVN was involved in both the parvocellular and magnocellular regions according to immunohistochemical analysis. Microinjection of asprosin into the PVN produced dose-related increases in RSNA, MAP, and HR, which were abolished by superoxide scavenger tempol, antioxidant N-acetylcysteine (NAC), and NADPH oxidase inhibitor apocynin. The asprosin promoted superoxide production and increased NADPH oxidase activity in the PVN. Furthermore, it increased the cAMP level, adenylyl cyclase (AC) activity, and protein kinase A (PKA) activity in the PVN. The roles of asprosin in increasing RSNA, MAP, and HR were prevented by pretreatment with AC inhibitor SQ22536 or PKA inhibitor H89 in the PVN. Microinjection of cAMP analog db-cAMP into the PVN played similar roles with asprosin in increasing the RSNA, MAP, and HR, but failed to further augment the effects of asprosin. Pretreatment with PVN microinjection of SQ22536 or H89 abolished the roles of asprosin in increasing superoxide production and NADPH oxidase activity in the PVN. These results indicated that asprosin in the PVN increased the sympathetic outflow, blood pressure, and heart rate via cAMP–PKA signaling-mediated NADPH oxidase activation and the subsequent superoxide production.

## 1. Introduction

Sympathetic activity plays a critical role in the regulation of blood pressure. Chronic excessive sympathetic activity contributes to the pathogenesis of hypertension, chronic heart failure, and chronic kidney disease [1,2,3,4]. Renal denervation is effective in lowering blood pressure in patients with resistant hypertension [5]. The hypothalamic paraventricular nucleus (PVN) is an important central integrative region in the regulation of cardiovascular activity and a major source of excitatory drive for sympathetic outflow to the spinal cord by both direct and indirect projections [6]. The excessive sympathetic activation in hypertension and chronic heart failure is mainly related to the changes in molecular signaling in the PVN [7,8].

Asprosin, a novel adipokine, was firstly identified in 2016 as a fasting-induced protein hormone that regulates hepatic glucose release [9]. The proprotein of asprosin is 2871 amino acids long and is cleaved by the activated protease furin at its C terminus to produce a 140-amino-acid-long asprosin and mature fibrillin-1 (FBN1) [10]. Asprosin contributes to metabolism and metabolic disorders, including obesity, diabetes, cardiovascular diseases, and polycystic ovary syndrome [11,12,13,14]. Asprosin can cross the blood–brain barrier to enhance the activity of orexigenic agouti-related peptide (AgRP) neurons in the hypothalamus to increase appetite [15]. Asprosin is widely distributed in multiple tissues and organs, including the hypothalamus of the brain [16]. Our preliminary study showed that abundant asprosin existed in the PVN. However, it is not known whether asprosin in the PVN is involved in regulating sympathetic activity and blood pressure.

Activation of the cAMP–protein kinase A (PKA) pathway in the PVN increases sympathetic outflow [17,18,19]. The classical cAMP–PKA signaling contributes to the reactive oxygen species (ROS) production induced by the β-adrenergic receptor agonist isoproterenol in mouse cardiomyocytes [20,21]. NADPH-oxidase-derived superoxide production in the PVN increases sympathetic outflow and mediates the angiotensin II or salusin-β-induced sympathetic activation [22,23,24]. The increased superoxide production in the PVN contributes to sympathetic overactivity in hypertension and chronic heart failure [7]. It was found that the cAMP–PKA pathway is necessary for asprosin-mediated AgRP neuron activation in the brain [15]. We were interested in whether cAMP and ROS production were involved in the effects of asprosin. Here we investigated the roles and underlined the mechanisms of asprosin in the PVN when regulating sympathetic activity and blood pressure.

## 2. Results

### 2.1. Asprosin Expression

**Figure 1:**
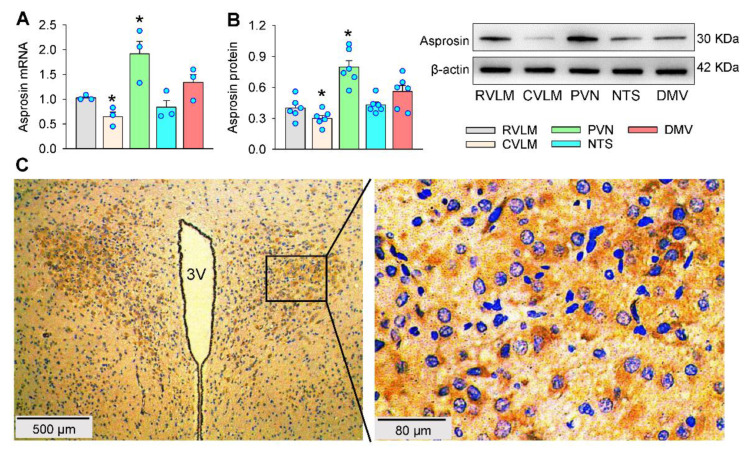
Asprosin expression in the PVN. (**A**) Relative asprosin mRNA levels in some nuclei of the brain. (**B**) Relative asprosin protein levels in some nuclei of the brain. (**C**) Representative images of immunohistochemistry for asprosin (brown color) in the PVN. The nuclei were stained with hematoxylin (blue color). * *p* < 0.05 vs. RVLM. Values are given as mean ± SE. *n* = 3 per group. RVLM, rostral ventrolateral medulla; CVLM, caudal ventrolateral medulla; PVN, paraventricular nucleus of hypothalamus; NTS, nucleus tractus solitaries; DMV, dorsal motor nucleus of the vagus; 3V, the third ventricle.

The asprosin expressions were examined and compared in several important nuclei of the brain for the regulation of sympathetic outflow and blood pressure. The asprosin mRNA level was higher in the PVN but lower in the caudal ventrolateral medulla (CVLM) compared with that in the rostral ventrolateral medulla (RVLM) (Figure 1A). Western blot analyses showed high asprosin expression in the PVN; moderate expression in the RVLM, nucleus tractus solitaries (NTS), and dorsal motor nucleus of the vagus (DMV); and low expression in the CVLM (Figure 1B). Immunohistochemistry for asprosin at the PVN level of the brain showed that high asprosin expression existed in the PVN, including both the magnocellular and parvocellular neurons in the PVN (Figure 1C). These results suggested a possibility that asprosin may have played crucial roles in the control of the sympathetic outflow and blood pressure.

### 2.2. Dose Effects and Time Effects of Asprosin in PVN

**Figure 2:**
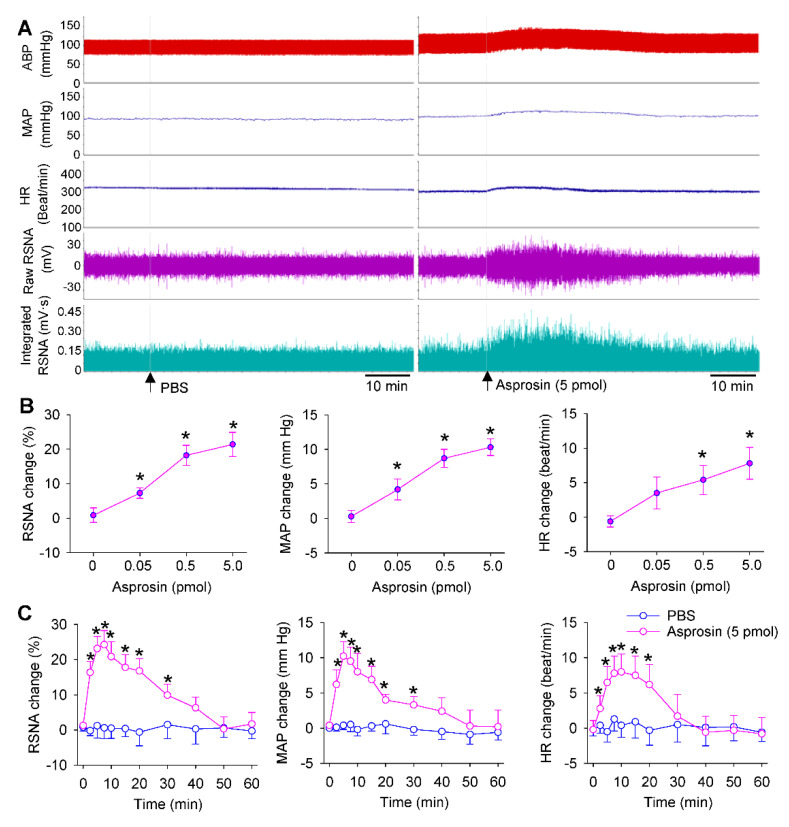
Effects of the microinjection of asprosin in the PVN on the RSNA, MAP and HR. (**A**) Representative recordings showing the effects of microinjection of PBS and asprosin (5 pmol) in the PVN. (**B**) Dose effects of asprosin (0, 0.05, 0.5, 5 pmol) in the PVN. * *p* < 0.05 vs. 0 pmol. (**C**) Time effects of microinjection of PBS and asprosin (5 pmol) in the PVN. * *p* < 0.05 vs. PBS. Values are given as mean ± SE. *n* = 6 per group.

Bilateral microinjection of asprosin in the PVN caused an immediate increase in the RSNA, MAP, and HR (Figure 2A). Asprosin dose-dependently increased the RSNA, MAP, and HR, and 5 pmol of asprosin almost reached its maximal effects (Figure 2B). The effects of asprosin lasted about 30 min, and the maximal effects occurred approximately 5 min after the PVN microinjection of asprosin. The PVN microinjection of PBS had no significant effects on the RSNA, MAP, and HR (Figure 2C).

### 2.3. Effects of Asprosin Antibody in the PVN

**Figure 3:**
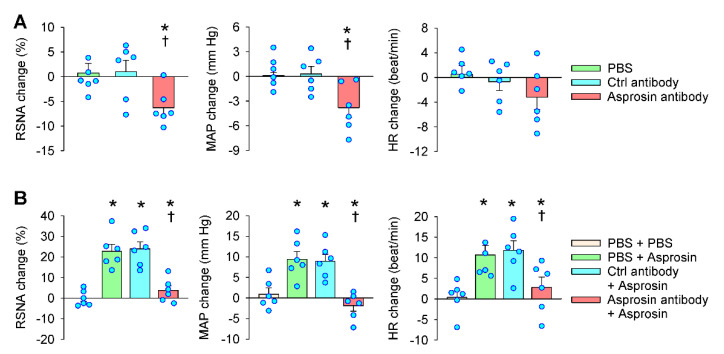
Roles of endogenous asprosin in regulating the RSNA, MAP, and HR. (**A**) Effects of microinjection of anti-asprosin antibody in the PVN on the RSNA, MAP, and HR. * *p* < 0.05 vs. PBS. † *p* < 0.05 vs. control antibody. (B) Effects of PVN pretreatment of anti-asprosin antibody on the roles of asprosin in increasing the RSNA, MAP, and HR. The pretreatment was carried out 10 min before the microinjection of asprosin (5 pmol) in the PVN. * *p* < 0.05 vs. PBS + PBS; † *p* < 0.05 vs. control antibody + asprosin. Values are given as mean ± SE. *n* = 6 per group.

Microinjection of specific antibodies in the PVN is a common method that is used to investigate the effects of endogenous active peptides [25,26,27]. The PVN microinjection of an anti-asprosin antibody to neutralize the endogenous asprosin reduced the RSNA and MAP (Figure 3A) and abolished the asprosin-induced increases in the RSNA, MAP, and HR (Figure 3B). The PVN microinjection of the control antibody had no significant effects. These results suggest that endogenous asprosin in the PVN played a tonic role in enhancing the sympathetic outflow.

### 2.4. Roles of Superoxide Production Mediated the Effects of Asprosin

**Figure 4:**
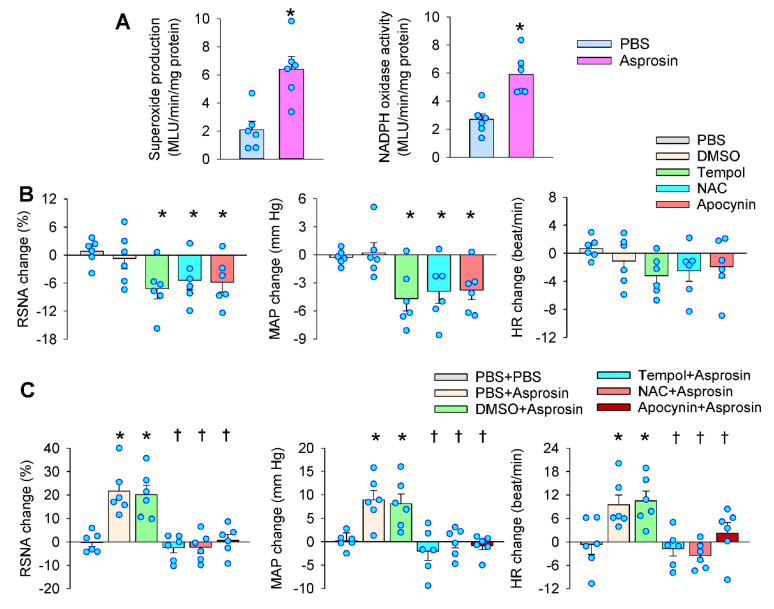
Roles of superoxide production in mediating the effects of asprosin in the PVN on the RSNA, MAP, and HR. (**A**) Effects of PVN microinjection of asprosin on superoxide production and NADPH oxidase activity in the PVN. The measurements were made 15 min after the microinjection. * *p* < 0.05 vs. PBS. (**B**) Effects of superoxide scavenger tempol (20 nmol), antioxidant NAC (40 nmol), and NADPH oxidase inhibitor apocynin (1 nmol) on the RSNA, MAP, and HR. * *p* < 0.05 vs. PBS or DMSO. (**C**) Effects of PVN pretreatment with tempol, NAC, and apocynin on the roles of asprosin in increasing the RSNA, MAP, and HR. The pretreatment was carried out 10 min before the microinjection of asprosin (5 pmol) in the PVN. * *p* < 0.05 vs. PBS + PBS; † *p* < 0.05 vs. PBS + asprosin or DMSO + asprosin. Values are given as mean ± SE. *n* = 6 per group.

Enhanced superoxide production in the PVN increases the sympathetic outflow [7]. We were interested to know whether superoxide production was involved in the effects of asprosin in the PVN. The microinjection of asprosin in the PVN increased superoxide production and NADPH oxidase activity (Figure 4A). The PVN microinjection of superoxide scavenger tempol, antioxidant N-acetylcysteine (NAC), or NADPH oxidase inhibitor apocynin reduced the RSNA, MAP, and HR (Figure 4B). Pretreatment with tempol, NAC, or apocynin in the PVN almost abolished the effects of asprosin in the PVN (Figure 4C). These results suggested that NADPH-oxidase-dependent superoxide production mediated the effects of asprosin in increasing the RSNA, MAP, and HR.

### 2.5. Roles of cAMP–PKA Signaling in Mediating the Effects of Asprosin in the PVN

**Figure 5:**
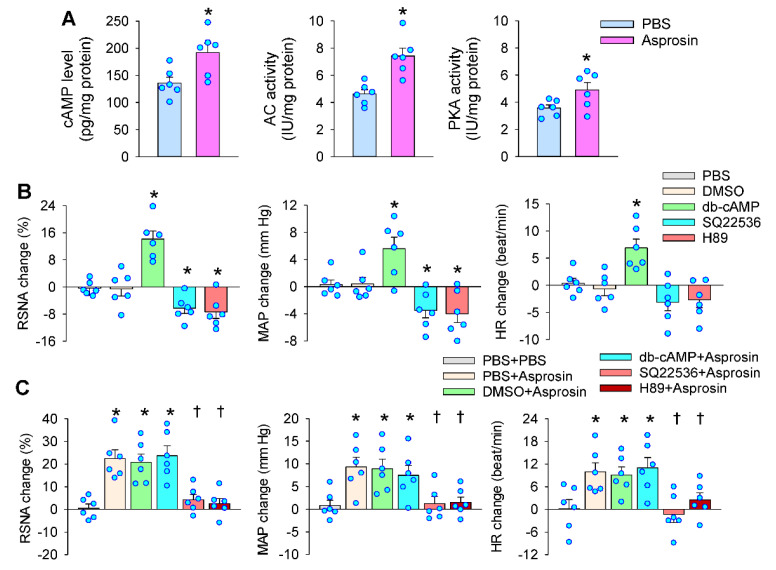
Roles of cAMP signaling in the effects of asprosin in the PVN on the RSNA, MAP, and HR. (**A**) Effects of PVN microinjection of asprosin on the cAMP levels, adenylyl cyclase (AC) activity, and protein kinase A (PKA) activity in the PVN. The measurements were made 15 min after the microinjection. * *p* < 0.05 vs. PBS. (**B**) Effects of the cAMP analog db-cAMP (1 nmol), AC inhibitor SQ22536 (2 nmol), and PKA inhibitor H89 (1 nmol) on the RSNA, MAP, and HR. * *p* < 0.05 vs. PBS or DMSO. (**C**) Effects of PVN pretreatment with db-cAMP, SQ22536, and H89 on the roles of asprosin in increasing the RSNA, MAP, and HR. The pretreatment was carried out 10 min before the microinjection of asprosin (5 pmol) in the PVN. * *p* < 0.05 vs. PBS + PBS; † *p* < 0.05 vs. PBS + asprosin or DMSO + asprosin. Values are given as mean ± SE. *n* = 6 per group.

The activation of cAMP–PKA pathway in the PVN promotes sympathetic activation [17,18,19]. The microinjection of asprosin into the PVN increased the cAMP level, AC activity, and PKA activity in the PVN (Figure 5A). The PVN microinjection of the cAMP analog dibutyryl-cAMP (db-cAMP) increased the RSNA and MAP, but AC inhibitor SQ22536 or PKA inhibitor H89 reduced the RSNA and MAP (Figure 5B). Pretreatment with SQ22536 or H89 in the PVN abolished the roles of asprosin, but db-cAMP failed to further enhance the roles of asprosin in increasing the RSNA, MAP, and HR (Figure 5C). These results indicated that asprosin-induced sympathetic activation was mediated by the cAMP–PKA signaling pathway in the PVN, which was supported by findings showing that the cAMP–PKA pathway contributes to asprosin-mediated glucose release in the liver [9] and AgRP neuron activation in the brain [15].

### 2.6. cAMP–PKA Signaling Mediated the Effects of Asprosin on Superoxide Production

**Figure 6:**
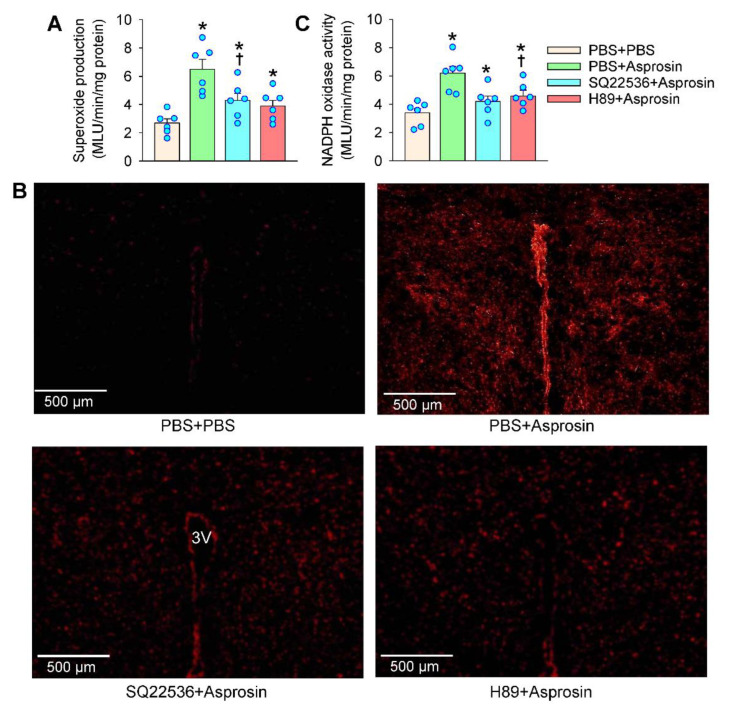
Effects of adenylyl cyclase inhibitor SQ22536 and PKA inhibitor H89 on the asprosin-induced changes in superoxide production and NADPH oxidase activity in the PVN. (**A**) Superoxide production. (**B**) Representative images showing the dihydroethidium (DHE) staining in the PVN. 3V, the third ventricle. (**C**) NADPH oxidase activity. The pretreatment with SQ22536 (2 nmol) or H89 (1 nmol) was carried out 10 min before the microinjection of asprosin (5 pmol) in the PVN. The measurements were made 15 min after the microinjection of asprosin. * *p* < 0.05 vs. PBS + PBS; † *p* < 0.05 vs. PBS + asprosin. Values are given as mean ± SE. *n* = 6 per group.

The cAMP–PKA signaling contributes to ROS production [20,21]. ROS plays a critical role in the cAMP-induced activation of Ras in Leydig cells [28]. We further examined whether cAMP–PKA signaling was involved in the NADPH oxidase activation and superoxide production in the PVN. The microinjection of AC inhibitor SQ22536 or PKA inhibitor H89 attenuated the asprosin-induced superoxide production in the PVN (Figure 6A). The role of SQ22536 or H89 in inhibiting the asprosin-induced superoxide production was involved in both the magnocellular and parvocellular neurons in the PVN (Figure 6B). Similarly, SQ22536 or H89 attenuated the asprosin-induced NADPH oxidase activation in the PVN (Figure 6C). These results indicated that the cAMP–PKA signaling pathway at least partially mediated the asprosin-induced NADPH oxidase activation and superoxide production.

## 3. Discussion

**Figure 7:**
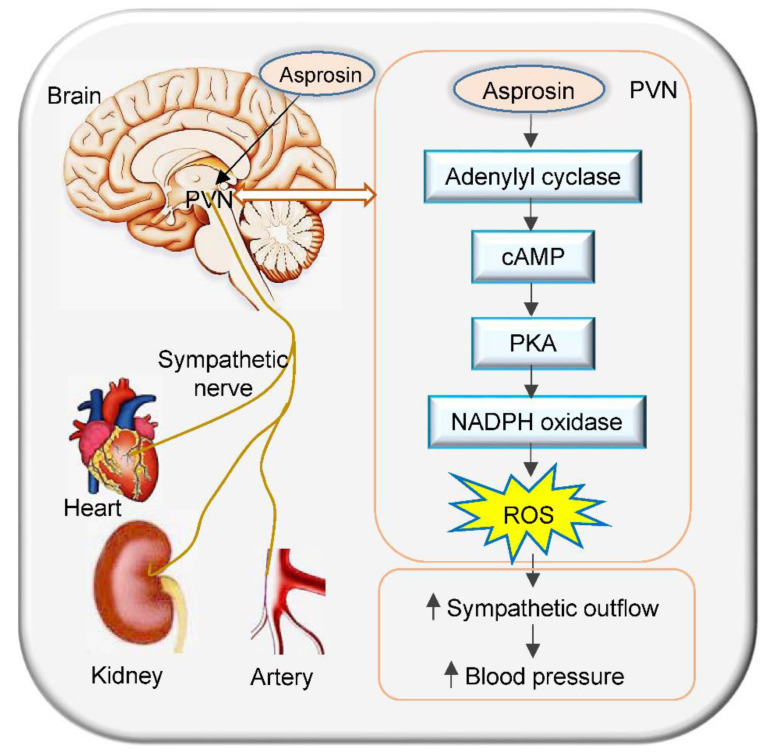
Schematic diagram showing the effects of asprosin in the PVN on the sympathetic outflow and blood pressure in rats and its signaling pathway.

Asprosin is an adipokine that is associated with metabolism and metabolic disorder [9]. It serves as a hormone that acts on the hypothalamus to increase appetite by crossing the blood–brain barrier [15]. The primary novel findings in this study were that asprosin in the PVN increased the sympathetic activity, blood pressure, and heart rate via NADPH-oxidase-dependent superoxide production. The cAMP–PKA signaling pathway was involved in the asprosin-induced NADPH oxidase activation and subsequent superoxide production (Figure 7).

The PVN, which is composed of parvocellular neurons and magnocellular neurons, is an important integrative site in the brain. The parvocellular neurons project to the intermediolateral cell column (IML) of the spinal cord and the RVLM neurons project to the IML and contribute to the regulation of sympathetic activity and blood pressure [29]. Magnocellular neurons express a variety of neuropeptides, including arginine vasopressin and oxytocin, and are involved in the regulation of both hydromineral homeostasis and autonomous functions [30]. The asprosin mRNA and protein expressions existed in several important nuclei in the brain for autonomic regulation, including RVLM, CVLM, PVN, NTS, and DMV, while the highest asprosin expression was found in the PVN. The asprosin-positive neurons included both parvocellular neurons and magnocellular neurons. The PVN microinjection of asprosin increased the RSNA, MAP, and HR, while neutralization of asprosin in the PVN with asprosin antibody reduced the RSNA, MAP, and HR. It was noted that asprosin induced an immediate increase in the RSNA and MAP with similar durations. According to the immediate response, the change range, and the parallel changes of the RSNA and MAP, the asprosin-induced pressor response was primarily caused by sympathetic activation. However, we cannot completely exclude the possibility that vasopressin might play a small role in the asprosin-induced pressor response. Persistent sympathetic activation not only increases blood pressure directly, and the increased norepinephrine from the sympathetic endings promotes extracellular vesicle release from adventitial fibroblasts of arteries [31]. These extracellular vesicles further contribute to vascular remodeling in hypertension [32,33].

Superoxide production in the PVN increased the sympathetic outflow [34]. We found that the microinjection of asprosin in the PVN increased the NADPH oxidase activity and superoxide production. Inhibiting NADPH oxidase or scavenging superoxides in the PVN not only reduced the RSNA, MAP, and HR but also abolished the effects of asprosin in the PVN. These results provided solid evidence that the roles of asprosin in the PVN in increasing the RSNA, MAP, and HR were mediated by NADPH-oxidase-dependent superoxide production. Previous studies showed that the increased superoxide production in the PVN attributes to the excessive sympathetic activation in hypertension [35], chronic heart failure [36], obesity [37], diabetes [38], and metabolic syndrome [23]. It is worthy to study the roles of asprosin in the PVN in the sympathetic over-activation of these diseases.

The cAMP–PKA signaling pathway in the PVN mediates the roles of angiotensin-(1-7) and alamandine in increasing the sympathetic activity [17,18,19]. Asprosin stimulates glucose release from hepatic cells via activating the cAMP–PKA pathway [39]. We found that the microinjection of asprosin into the PVN increased the cAMP level, AC activity, and PKA activity in the PVN. The inhibition of AC or PKA in the PVN reduced the RSNA and MAP and abolished the roles of asprosin in increasing the RSNA, MAP, and HR. The cAMP analog db-cAMP increased the RSNA and MAP but did not further enhance the roles of asprosin. These results indicated that the cAMP–PKA signaling pathway in the PVN mediated the asprosin-induced sympathetic activation. The inhibition of AC or PKA in the PVN attenuated the asprosin-induced NADPH oxidase activation and superoxide production in the PVN. These results indicated that cAMP–PKA signaling mediated the asprosin-induced superoxide production, which was supported by the findings that superoxides contribute to the cAMP-induced activation of Ras in Leydig cells [28]. It was noted that the inhibition of AC or PKA in the PVN could not completely abolish the effects of asprosin on the NADPH oxidase activity and superoxide production, suggesting that there might be some other signals involved in mediating the asprosin-induced NADPH oxidase activity and superoxide production. It was found that serum asprosin is positively related to angiotensin-converting enzyme inhibitor/angiotensin II receptor blocker therapy in type 2 diabetes patients [40]. However, the interaction of asprosin with angiotensin in the PVN is not known. Our previous studies showed that angiotensin II in the PVN induces sympathetic activation and pressor responses via NADPH-oxidase-derived superoxide production [36], and that Ang-(1-7) contributes to the enhanced sympathetic outflow via the cAMP–PKA pathway in renovascular hypertension [17]. In the present study, we found that asprosin in the PVN increased the sympathetic outflow, blood pressure, and heart rate via cAMP–PKA signaling-mediated NADPH oxidase activation and subsequent superoxide production. These results suggested that the interaction of asprosin with the angiotensin system might exist in their downstream signaling pathway, which needs further investigation.

A previous study showed that asprosin promotes the upregulation of the antioxidant enzyme superoxide dismutase 2 (SOD2) in mesenchymal stromal cells and that asprosin inhibits the hydrogen-peroxide-induced ROS generation and apoptosis via ERK1/2-SOD2 pathway in these cells [41]. This study showed the novel mechanism of asprosin. We found that asprosin induced an immediate sympathetic activation and increased NADPH oxidase activity and superoxide production. The rapid responses must be mediated by the intracellular second messenger rather than SOD2 or other anti-oxidant enzyme expressions; therefore, we did not measure the antioxidant enzyme expressions in the PVN in this study. However, we could not exclude the possibility that long-term asprosin administration may increase the expressions of antioxidant enzymes in the PVN via its direct or secondary mechanism. The different roles of asprosin in modulating the ROS may attribute to the different receptors. So far, three types of asprosin receptors have been reported as follows: (1) asprosin promoted glucose release in the liver through its G-protein-coupled receptor called OR4M1 and its downstream cAMP–PKA pathway [9], (2) the olfactory receptor OLFR734 acted as an asprosin receptor to regulate hepatic glucose production [42] and male fertility [43], and (3) asprosin impairs insulin secretion through toll-like receptor 4 (TLR4) and its downstream JNK-mediated inflammation [44]. Asprosin activated cAMP–PKA pathway to cause sympathetic activation in the present study. We speculate that the effects of asprosin in the PVN may be mediated by OR4M1 or OLFR734 receptors. A limitation of the present study was that the exact receptors of asprosin in the PVN in modulating sympathetic outflow were not identified because specific antagonists of OR4M1 or OLFR734 receptors are not available at present.

## 4. Materials and Methods

### 4.1. Animals and General Procedures

Experiments were performed in male SD rats weighing between 300 and 350 g. The rats were kept in an environment under a 12 h cycle of light/dark with controlled temperature and humidity. Standard laboratory chow and tap water were available ad libitum. Rats were anesthetized with an intraperitoneal injection of a mixture of urethane (800 mg/kg) and α-chloralose (40 mg/kg). The animal was kept in a supine position and the trachea and carotid artery were exposed through a vertical incision in the middle of the neck. Endotracheal intubation was made and connected to a small animal ventilator (683, Harvard Apparatus Inc., Holliston, MA, USA) for positive pressure ventilation with room air. A PE50 catheter connected with a pressure transducer was inserted into the right carotid artery for the blood pressure recording. Then, the rats remained in a prone position and fixed on a stereotaxic frame (Stoelting, Chicago, IL, USA) to perform surgery for the PVN microinjection and the renal sympathetic nerve activity (RSNA) recording. Both the blood pressure and RSNA signals were recorded with a data acquisition system (8SP, ADInstruments, Bella Vista, NSW, Australia). The rats were stabilized for at least 30 min before the experiment and finally euthanized via intravenous injection of sodium pentobarbital (100 mg/kg).

### 4.2. RSNA Recording

A left retroperitoneal incision was made to expose the left renal nerve. The nerve was isolated and cut at its distal end to abolish the renal afferent activity. The central end of the nerve was put on a pair of platinum electrodes and immersed in mineral oil at 37 °C. The nerve signals were amplified using a model DP-304 differential amplifier (Warner Instruments, Hamden, CT, USA). The band-pass filtration was set at 100–3000 Hz. The RSNA was integrated at a time constant of 100 ms with LabChart 8 software (ADInstruments, Bella Vista, NSW, Australia). Background noise was obtained by cutting the central end of the nerve. The percentage change of integrated RSNA from the baseline value was calculated after each intervention [25].

### 4.3. PVN Microinjection

Stereotaxic coordinates for the PVN microinjection were 0.4 mm lateral to the midline, 1.8 mm caudal from bregma, and 7.9 mm ventral to the dorsal surface according to the Paxinos and Watson rat atlas. Bilateral PVN microinjections were made through glass micropipettes (tip outer diameter 50 µm) with a 0.5 µL microsyringe and completed within 1 min. The microinjection volume for each side of the PVN was 50 nL. In the end, the same volume of Evans Blue was microinjected into the PVN and prepared for the histological identification of the microinjection sites. The rats were excluded if the microinjection site was out of the PVN. A total of 6 rats were excluded from the data analysis in the present study.

### 4.4. In Situ Detection of the Superoxide Level in the PVN

DHE (Beyotime Biotechnology, Shanghai, China) was used as a specific fluorogenic probe for detecting superoxide levels in the PVN [26]. The rats were euthanized and the brains were rapidly removed, frozen with liquid nitrogen, embedded into the tissue OCT-Freeze Medium, and cryostat-sectioned (30 µm, coronal) onto the chilled microscope slides. The sections were thawed at room temperature, rehydrated with phosphate-buffered saline, and incubated for 5 min in the dark with DHE (1 µmol/L). After washing with phosphate-buffered saline, the DHE fluorescence in sections was detected with a fluorescence microscope (BX51, Olympus, Tokyo, Japan) with an excitation wavelength of 543 nm. The detector and laser settings were kept constant among all samples.

### 4.5. Measurements of Superoxide Production and NADPH Oxidase Activity

Coronal sections with a thickness of 450 µm at the PVN level were performed with a cryostat microtome (Model CM1900, Leica, Wetzlar, Germany). The PVN tissue was punched out with a 15-gauge needle, homogenized in a lysis buffer, and then centrifuged. The total protein concentration was measured with Bradford assay kit (BCA; Pierce, Santa Cruz, CA, USA). The lucigenin-derived chemiluminescence method was used to measure superoxide production and NADPH oxidase activity in the PVN [27,28]. Photon emission was triggered by adding dark-adapted lucigenin (5 μM) to measure the superoxide production, or both dark-adapted lucigenin (5 μM) and NADPH (100 μM) to measure the NADPH oxidase activity. Light emission was measured with a luminometer (Model 20/20n, Turner, CA, USA) ten times in 10 min. Background chemiluminescence was measured in the buffer containing lucigenin (5 μM). The data were expressed in terms of the mean light unit (MLU)/min/mg protein.

### 4.6. Quantitative PCR

Total RNA in the RVLM, CVLM, PVN, NTS, and DMV of the rat was extracted with the reagent (Life Technologies, Gaithersburg, MD, USA). The purity and concentration of the extracted RNA were determined with a UV spectrophotometer. The RNA samples were subjected to a reverse transcription reaction to synthesize cDNA templates, which were then subjected to PCR amplification. According to the consistency of the standard curve amplification efficiency, the relative expression levels of asprosin mRNA in the samples were analyzed using the ΔΔCt method. GAPDH was used as a normalized control. The primers used are listed in the online Supplementary Materials (Table S1).

### 4.7. Western Blot

Asprosin protein expression in the RVLM, CVLM, PVN, NTS, and DMV was examined with Western blotting. Equal amounts of protein extracts were electrophoresed on 10% SDS-PAGE and transferred onto a PVDF membrane. After blocking with 5% milk, the membranes were incubated with the antibody against asprosin (1:1000) overnight at 4 °C followed by incubation in HRP-conjugated secondary antibody (1:5000) for 1 h at room temperature. The blots were visualized with chemiluminescence. β-actin was employed as a normalized control.

### 4.8. Measurements with Commercial Kits

Measurements were performed following the manufacturer’s descriptions of the kits. The cAMP content was measured with a Cyclic Adenosine Monophosphate Assay Kit (Nanjing Jiancheng Bioengineering Institute, Nanjing, China). The AC activity was examined with Adenylate Cyclase Activity Assay Kit (Mlbio Co., Shanghai, China). PKA activity was determined with PKA Activity Kit (Enzo Life Sciences, Ann Arbor, MI, USA). Absorbance was examined at 450 nm using an automatic plate reader (ELx800, Biotek Instruments, Winooski, VT, USA).

### 4.9. Immunohistochemistry

Immunohistochemistry was performed to detect the asprosin expression in the brain sections at the PVN level of rats. The primary anti-asprosin antibody was obtained from FineBiotechCo. (Wuhan, China) and was diluted for use (1:200). Horseradish peroxidase-conjugated goat anti-rabbit antibody was acquired from Santa Cruz Biotechnology Inc. (Santa Cruz, CA, USA). Images were taken with a light microscope (BX-51, Olympus, Tokyo, Japan).

### 4.10. Chemicals

SQ22536, db-cAMP, H89, and asprosin were purchased from Med Chem Express (Monmouth Junction, NJ, USA). Tempol, apocynin, and NAC were obtained from Sigma (St. Louis, MO, USA). Asprosin and tempol were dissolved in PBS, and other chemicals were dissolved in PBS containing 1% DMSO. Vehicles were used as controls.

### 4.11. Statistics

RSNA, MAP, and HR values were assessed by averaging 1 min data. All data were expressed as mean ± SE. Student’s *t*-test was used to evaluate the statistical significance of the difference between the two groups. Multiple comparisons were performed using one-way or two-way ANOVA followed by Bonferroni’s post hoc analysis. A *p*-value < 0.05 was considered statistically significant.

## 5. Conclusions

Asprosin in the PVN increased the sympathetic outflow, blood pressure, and heart rate. The effects of asprosin were mediated by the cAMP–PKA signaling-pathway-mediated NADPH oxidase activation and subsequent superoxide production.

## Data Availability

Data for this study are available from the corresponding author on reasonable request.

## Supplementary Materials

The following supporting information can be downloaded from https://www.mdpi.com/article/10.3390/ijms232012595/s1.
