# Supplementary material for: Asprosin in the Paraventricular Nucleus Induces Sympathetic Activation and Pressor Responses via cAMP-Dependent ROS Production
Source: Int J Mol Sci. 2022 Oct 20;23(20):12595. doi: 10.3390/ijms232012595 (PMC9604496; doi:10.3390/ijms232012595)
Supplement: Supplementary file 1 [file ijms-23-12595-s001.zip › ijms-1960700-supplementary.pdf]

Online Data Supplement

Table S1. Primers for qPCR analysis

Name	Primer	Sequence
Asprosin	Forward	5’-CGAATCCTAGAGCTCCTGCC-3’
	Reverse	5’-GGAGGTAGCTGACCCCTTCT-3’
GAPDH	Forward	5’-GGGTGTGAACCACGAGAAAT-3’
	Reverse	5’-ACTGTGGTCATGAGCCCTTC-3’
